# Clinical Features and Outcomes of Patients with Full Spectrum of COVID-19 Severity and Concomitant Herpesvirus Reactivation

**DOI:** 10.3390/microorganisms13061221

**Published:** 2025-05-27

**Authors:** Paolo Ravanini, Maria Grazia Crobu, Claudia Martello, Giulia Faolotto, Luigi Mario Castello, Antonia Palumbo, Luigi Maria Fenoglio, Clotilde Impaloni, Melissa Briasco, Christian Di Domenico, Paola Macaluso, Alessio Mercandino, Miriam Riggi, Mario Pirisi, Stefano Andreoni, Carlo Smirne

**Affiliations:** 1 Laboratory of Microbiology and Virology, Maggiore della Carità Hospital, 28100 Novara, Italy; paolo.ravanini@maggioreosp.novara.it (P.R.); mcrobu@cittadellasalute.to.it (M.G.C.); claudiamartello14@gmail.com (C.M.); giulia.faolotto@maggioreosp.novara.it (G.F.); antonia.palumbo@maggioreosp.novara.it (A.P.); clotildeimpaloni@gmail.com (C.I.); melissa.briasco@maggioreosp.novara.it (M.B.); christian.didomenico@maggioreosp.novara.it (C.D.D.); paola.macaluso@maggioreosp.novara.it (P.M.); alessio.mercandino@maggioreosp.novara.it (A.M.); miriam.riggi@maggioreosp.novara.it (M.R.); stefano.andreoni@maggioreosp.novara.it (S.A.); 2 Clinical Biochemistry Laboratory, Department of Laboratory Medicine, AOU Città della Salute e della Scienza di Torino, 10126 Turin, Italy; 3 Internal Medicine Unit, SS. Antonio e Biagio e Cesare Arrigo University Hospital, 15121 Alessandria, Italy; luigi.castello@med.uniupo.it; 4 Department of Translational Medicine, Università del Piemonte Orientale, 28100 Novara, Italy; mario.pirisi@med.uniupo.it; 5 Department of Internal Medicine, Santa Croce e Carle Hospital, 12100 Cuneo, Italy; fenoglio.l@ospedale.cuneo.it; 6 Internal Medicine Unit, Maggiore della Carità Hospital, 28100 Novara, Italy

**Keywords:** COVID-19, SARS-CoV-2, human herpesviruses, herpes simplex virus 1, Epstein–Barr virus, human herpesvirus 6, human herpesvirus 7, human cytomegalovirus, viral reactivation, coinfection

## Abstract

Some studies suggested a high incidence of human herpesvirus (HHV) reactivation in severe acute respiratory syndrome coronavirus 2 (SARS-CoV-2) infection. To evaluate the prevalence of HHV reactivations in a population with various severity degrees of coronavirus disease 2019 (COVID-19), we analyzed 102 individuals and compared them with 51 SARS-CoV-2-negative subjects admitted in the same period (January–July 2022) for acute respiratory failure. Positivity was found in 76% of subjects for at least one HHV, and in 46% for ≥2 HHV. These proportions were more prevalent in SARS-CoV-2-positive than in negative patients (83% vs. 61%; 56% vs. 27%, respectively). The most common HHV was HHV-7 both in the whole population (51%) and in SARS-CoV-2-positive and -negative subjects (57% and 39%, respectively); human cytomegalovirus, herpes simplex virus-1, Epstein–Barr virus, and HHV-6 were more represented in SARS-CoV-2-positive individuals. No single or combined HHV reactivation was associated with the 60-day mortality rate. However, cytomegalovirus reactivation was an independent predictor of COVID-19 severity and longer hospitalizations, while the occurrence of ≥3 any HHV reactivations was independently associated with the aforementioned outcomes and ventilatory support need. Taken together, our data suggest that in patients with moderate-to-severe COVID-19, the diagnosis of HHV coinfections can add useful prognostic information.

## 1. Introduction

The severe acute respiratory syndrome coronavirus 2 (SARS-CoV-2) pandemic has raged through the world from December 2019 [1,2]. The whole spectrum of coronavirus disease 2019 (COVID-19) includes asymptomatic, mild-to-moderate, and severe-to-critical illness [3,4]. In the latter case—which was particularly common during the first waves─ a severe respiratory distress syndrome can occur, requiring urgent hospitalization and, sometimes, mechanical ventilatory support. Despite aggressive treatment, the clinical course can then ultimately be followed by death due to the triggering of a phase known as “cytokine storm”, which, in turn, induces immunosuppression and leucopenia [3,4,5,6]. Several factors have been shown to exacerbate the clinical outcomes of COVID-19. These include underlying comorbidities, but also superadded bacterial, fungal, or viral infections. However, coinfections with other viruses are not well-studied at this time [7,8,9]. Specifically, adults harbor a variety of persistent viral infections that affect the generation of immune responses. In addition, reactivation of preexisting infections can influence the response to new ones, including COVID-19 [10].

One of the most common chronic infections in humans is caused by human herpesviruses (HHV). These pathogens share a latent phase but are capable of reactivation under the influence of a variety of stimuli, including immunosuppression, cellular stress, and tissue damage [11,12,13]. All of these events are expected in patients with COVID-19 due to both the damage caused by the disease and the treatments patients receive, including systemic steroids [14]. This occurrence is mainly common in critically ill patients, even when there is no history of pre-existing immunodeficiency [15,16]. Moreover, there is established evidence that also comorbid conditions can trigger these reactivations [17].

Taking into account that the majority of the population is infected with HHV, and that it is still under debate as to how their possible reactivation and their eventual treatment can alter the clinical course of COVID-19 patients, the aim of the present study was to investigate the cumulative incidence and impact of HHV reactivations in a large population of SARS-CoV-2-positive subjects presenting with the whole spectrum of the disease, trying to overcome the limitations presented by most of the so-far-published literature that is focused mainly on critically ill patients.

## 2. Materials and Methods

### 2.1. Study Design

This was a single-center, retrospective, and observational study on all consecutive human immunodeficiency virus (HIV)-negative patients admitted from January 2022 to July 2022 to Novara University Hospital (Italy) for COVID-19. The research also enrolled the following other subsets of individuals: (a) a population of non-consecutive patients admitted in the above-mentioned hospital for acute respiratory failure in the same time frame, but which was SARS-CoV-2-negative; (b) a group of asymptomatic health workers who tested positive for SARS-CoV-2 infection at routine screening during in the same period and willing to donate a swab sample; (c) a group of paucisymptomatic subjects found positive for SARS-CoV-2 in the emergency department in the same time frame, but without any respiratory failure or other major symptoms and, thus, discharged home. For the purposes of this study, (b) and (c) groups were considered together in a cumulative group called “asymptomatic/mild cases”.

All the aforementioned individuals underwent, as part of normal clinical practice, SARS-CoV-2 detection by nasopharyngeal molecular swab; these samples were then stored at −80 °C in the hospital microbiology laboratory until their reuse with the written consent of the patients or their next of kin (in the event of inability to give consent due to adverse clinical conditions or premature death). To note, none of the subjects enrolled in this study performed HHV detection during their initial hospital stay. The research was conducted in strict adherence to the principles of the Declaration of Helsinki of 1975, as revised in 2000. The study protocol was approved by the local institutional Ethics Committee (Comitato Etico Interaziendale Novara, https://comitatoetico.maggioreosp.novara.it/, IRB code CE097/2020, accessed on 21 April 2025).

The main study inclusion criteria were as follows: (a) available previous molecular diagnosis for SARS-CoV-2; (b) age ≥ 18 years; (c) availability of a sufficient amount of nasopharyngeal swab sample stocked at −80 °C at the local virology laboratory and correctly stored; (d) written informed consent as above specified; (e) available medical records and laboratory archives containing thorough clinical and laboratory information, with a maximum allowed latency time of 48 h from the first positive swab for SARS-CoV-2, and including follow-up data at 30 and 60 days after the first SARS-CoV-2 detection or hospital admittance for SARS-CoV-2-negative subjects.

### 2.2. Laboratory Methods

Samples used for the study were nasopharyngeal swabs using the Universal Transport Medium (UTM-RT^®^) System^TM^ (Copan Diagnostics, Murrieta, CA, USA). The routine assay used at that time in our laboratory for the molecular detection of SARS-CoV-2 RNA was the real-time reverse transcriptase (RT) polymerase chain reaction (PCR) test ALINITY m SARS-CoV-2 (Abbott Laboratories, Abbott Park, IL, USA).

For the purposes of this study, all nasopharyngeal swab samples were retested using a quantitative PCR (ELITechGroup, Puteaux, France) for the following targets: herpes simplex virus (HSV)-1 and HSV-2 (HSV 1&2 ELITe MGB^®^ kit), varicella-zoster virus (VZV) (VZV ELITe MGB kit), human cytomegalovirus (HCMV) (CMV ELITe MGB^®^ kit), Epstein–Barr virus (EBV) (EBV ELITe MGB^®^ kit), HHV-6 (HHV6 ELITe MGB^®^ kit), HHV-7 (HHV7 ELITe MGB^®^ kit), and HHV-8 (HHV8 ELITe MGB^®^ kit). HHV reactivation was defined as an HHV-positive RT-PCR in any respiratory sample.

### 2.3. Statistical Analysis

Continuous variables were summarized as medians and inter-quartile ranges (IQR), and categorical variables as frequencies and percentages. All data were assessed for normality using a Shapiro–Wilk test and quantile–quantile (Q–Q) plot observation. Continuous variables were compared between groups using the Kruskal–Wallis or the Mann–Whitney test, as appropriate. Categorical variables were compared across subgroups using the χ^2^ test, Pearson’s chi-squared test, Fisher’s exact test, or the χ^2^ test for linear trend (Cochran–Armitage test), as appropriate. The differences between independent groups in the presence of ordinal dependent variables from a continuous distribution were compared through the Mann–Whitney test (two groups) and the ANOVA or Kruskal–Wallis tests (more than two groups), as appropriate. Multivariate logistic regression analysis with stepwise forward selection was performed to evaluate the factors independently associated with COVID-19 severity and certain outcomes (need for ventilatory assistance, length of hospitalization, and occurrence of death events), with *p* values less than 0.05 as the criteria for model inclusion.

All *p* values are two-tailed and are considered statistically significant when <0.05. Analyses were performed using Stata 18 statistical software (StataCorp LLC, College Station, TX, USA).

## 3. Results

### 3.1. Characteristics of the Study Population

For this study, 389 individuals were initially evaluated. The following subjects were excluded after applying the aforementioned criteria: lack of valid written informed consent or withdrawal of consent (*n* = 86); lack of a residual sufficient amount of swab sample (*n* = 68); lack of available complete medical records including the predefined follow-up (*n* = 82). Ultimately, 153 Caucasian subjects were selected for this study: 51 were SARS-CoV-2-negative, while the remaining 102 were SARS-CoV-2-positive. The latter ones were differently managed according to the severity of their disease and the presence of recognized baseline risk factors (such as age over 60 years, hypertension, diabetes, cardiovascular disease, chronic respiratory disease, and immunocompromising conditions), according to the local diagnostic and therapeutic care pathway and current evidence available at that time [18]. Specifically, (a) asymptomatic/mild cases (*n* = 28/6, respectively; total *n* = 34) underwent hospital discharge with quarantine and self-monitoring at home; of the latter subgroup, one subject was orally treated with molnupiravir and three with nirmatrelvir/ritonavir, as available in Italy at that time for non- hospitalized COVID-19 patients without respiratory failure; (b) moderately severe cases (*n* = 32) who required supplemental oxygen administration, including high flow nasal cannula (HFNC), continuous positive airway pressure (CPAP), or non-invasive ventilation (NIV) support, were admitted to internal medicine or to other medical wards with low- and medium-intensity care; (c) severe/critical cases (*n* = 36) were admitted to an intensive care unit (ICU) and could require intubation and mechanical ventilation. SARS-CoV-2-negative patients, in turn, were hospitalized in various non-COVID-19-dedicated medical units, generally internal medicine or respiratory wards, with ICU admittance in the most severe forms of respiratory failure. In all cases, if a patient during the same hospitalization was admitted to two or more wards, the one with the highest intensity of care was taken into account.

The main baseline clinical and demographical characteristics of the studied population are reported in Table 1. ICU-admitted patients were on average older (*p* = 0.016) and had a tendency to bear more comorbidities (*p* = 0.073); no significant differences could be found concerning sex distribution (*p* = 0.291). Focusing on SARS-CoV-2-positive individuals (*n* = 102), ICU-admitted ones (*n* = 36) showed, as expected, significant lower COVID-19 vaccination rates (*p* = 0.005). Additional clinical and laboratory parameters of the whole cohort of SARS-CoV-2-positive subjects are summarized in Table 2.

Briefly, though not being a randomized study, SARS-CoV-2-positive and -negative groups (2:1 ratio) were well balanced for most clinical and demographic variables. The main obvious difference consisted in that SARS-CoV-2-negative individuals had, on average, a more severe disease burden compared to SARS-CoV-2-positive ones, but this resulted from the fact that, as per protocol, the former subpopulation consisted exclusively of hospitalized patients in order to have higher probabilities of detecting HHV-positive cases. Instead, SARS-CoV-2-positive subjects also included asymptomatic/mild cases. However, when focusing on the proportions of SARS-CoV-2-positive and -negative patients with severe/critical respiratory failure, no significant differences between the two subgroups were evident (*p* = 0.718).

SARS-CoV-2-negative subjects were very similar to SARS-CoV-2-positive ones also for most pre-existing diseases, except for the prevalence of chronic lung disease, and consequently of COPD exacerbations, which were higher in the former group. As expected, SARS-CoV-2-positive subjects had significantly lower vaccination rates for COVID-19 than SARS-CoV-2-negative patients. For what concerns laboratory parameters, again they were comparable in most cases. The most relevant difference was that, as widely described, SARS-CoV-2-positive subjects had more severe COVID-related neutropenia. COVID-19 subjects had generally worse outcomes than SARS-CoV-2-negative ones (both for concerns mean hospital stay and death incidence), despite the fact they underwent appropriate medical treatments. As a matter of fact, they were administered antiviral therapy in almost all cases, as well as systemic corticosteroid therapy in those who in turn needed supplemental oxygen therapy (about 80% of this subpopulation), in accordance with the European Medicines’ Agency recommendations and the available literature at that time [22].

### 3.2. Analysis of Herpesvirus Reactivation(s)

The vast majority of the study population (116/153, 76%) tested positive for at least one HHV, and 71 (46%) showed coinfection with two or more viral subtypes. These two proportions were significantly more represented in the subset of SARS-CoV-2-positive subjects compared to SARS-CoV-2-negative ones. When considering only subjects with ≥3 HHV reactivations (*n* = 27), they all belonged to the SARS-CoV-2-positive subpopulation and were generally affected by the most severe forms of disease. Specifically, the numbers of individuals displaying three, four, and five HHV reactivations were 19, 7, and 1, respectively.

The most prevalent detected HHV was HHV-7, both in the whole study population (51%) and when considering SARS-CoV-2-positive and -negative subjects (57% and 39%, respectively), without significant differences between the latter two groups. HSV-1, EBV, and HHV-6 were more frequent in the SARS-CoV-2-positive subset of patients (respectively, 27% vs. 2%; 39% vs. 18%; 46% vs. 22%). Conversely, HHV-8 (though observed only in 2% of SARS-CoV-2-negative individuals) and HCMV did not show different proportions of reactivation between SARS-CoV-2-positive and -negative individuals. No reactivations for HSV-2 and VZV were observed in this study. All these data are detailed in Table 3. Further analyses on the prevalence of HHV reactivation(s) in other subsets of patients are reported in Supplementary Table S1, including the comparison between ICU- and non-ICU-admitted patients. Briefly, most HHV reactivations were confirmed to be prevalent in SARS-CoV-2-positive individuals (HSV-1, EBV, HHV-6), all the more so if they bore severe stages of disease (HSV-1, HCMV, HHV-6). Supplementary Figure S1 reports the radiological imaging of the single SARS-CoV-2-positive patient with all five HHV reactivations detected at the same time, as this is a paradigmatic example of a multifocal viral pneumonia causing severe acute respiratory failure (no history of pre-existing chronic lung disease in this subject) with an unfavorable outcome, despite prolonged maximal treatment in ICU.

Focusing on SARS-CoV-2-positive subjects (*n* = 102), no significant differences could be found in the median cycle threshold (Ct) values at SARS-CoV-2 RT-PCR between the individuals who experienced or did not experience HHV reactivation(s), both single and combined (Supplementary Table S2).

### 3.3. Association of Patients’ Characteristics with Herpesvirus Reactivation(s)

In order to further investigate which factors could be associated with HHV reactivations in SARS-CoV-2-positive individuals, we compared their main clinical and demographic characteristics—at baseline or during hospitalization, as appropriate—according to whether or not they had any viral reactivation (Table 4): panel A concerns HSV-1, HCMV, and EBV, while panel B is centered on HHV-6, HHV-7, and on any multiple (≥3) HHV reactivations.

In more detail, the most relevant finding that emerged from the comparison of subjects who had HHV reactivation with those who did not was that the former ones—while generally showing no significant differences with regard to the main biochemical variables with the partial exceptions of median lymphocyte count (HHV-6, HHV-7) and interleukin (IL)-6 levels (HSV-1, EBV)—had a major negative impact with regard to their clinical course. In fact, individuals with HHV reactivations were more likely to have severe forms of disease (HSV-1, HHV-6, HHV-7), require hospitalization in ICU (HSV-1, HCMV), and/or have longer durations of hospitalization (HSV-1, HCMV, HHV-6, HHV-7). Specifically, HHV coinfections showed their maximum effects when they were multiple (≥3): in those cases, all three of the unfavorable outcomes just described were more represented, and in addition, patients had a greater need for respiratory support. In turn, HHV reactivations were more likely in individuals undergoing systemic steroid therapies for concomitant COVID-19 and were generally associated with increased serum IL-6 levels. In this case series, HHV reactivations, either single or combined, did not prove to be associated with higher overall 30-day and 60-day mortality rates in SARS-CoV-2-positive patients, with the exception of EBV.

In order to exclude as a possible confounding factor, the fact that the study design included also non-hospitalized subjects (i.e., those with asymptomatic-to-mild disease, *n* = 34), the former analyses were repeated by considering only hospitalized subjects (i.e., those with moderate and severe-to-critical forms of COVID-19, *n* = 68). In this case, no differences were confirmed concerning mortality rates (EBV, *p* = 0.083; ≥3 HHV reactivations, *p* = 0.549), occurrence of severe forms of disease/need for hospitalization in ICU (HSV-1, *p* = 0.151; HHV-6, *p* = 0.778; HHV-7, *p* = 0.254), and occurrence of longer durations of hospitalization (HSV-1, *p* = 0.327; HHV-6, *p* = 0.778; HHV-7, *p* = 0.401). However, HCMV as a single reactivation confirmed its deleterious role in this subset of patients, both for what concerns the occurrence of the more severe forms of disease (*p* = 0.035) and the need for longer durations of hospitalization (*p* < 0.001). Similar observations could be made also for multiple (≥3) HHV reactivations, in that they were more represented in the subjects suffering from the more severe forms of COVID-19 (*p* = 0.035), requiring ventilatory support (*p* = 0.047), or needing longer lengths of hospitalization (*p* = 0.013).

### 3.4. Factors Associated with Patients’ Outcomes in SARS-CoV-2-Positive Patients

The following hard endpoints were analyzed in the cohort of SARS-CoV-2-positive subjects: (a) occurrence of severe/critical forms of illness resulting in ICU admission; (b) need for total hospital stay > 14 days; (c) need for any form of ventilatory assistance (both non-invasive and invasive); (d) occurrence of death within 60 days from the first SARS-CoV-2 positivity (Table 5). In order to avoid a possible selection bias due to the study design, again, only hospitalized patients (*n* = 68) were included in these analyses. Multivariate analysis—performed among those factors with *p* < 0.05 at univariate analysis—identified the following virological variables as possible independent predictors of the aforementioned adverse outcomes: for what concerns the occurrence of the severe/critical forms of COVID-19, HCMV reactivation (OR 3.914, *p* = 0.008), and ≥3 any HHV reactivations (OR 3.914, *p* = 0.008); regarding the occurrence of hospital stays > 14 days, HCMV reactivation (OR 3.914, *p* = 0.008), and ≥3 any HHV reactivations (OR 3.914, *p* = 0.008); with regard to the need for non-invasive or invasive ventilatory assistance, ≥3 any HHV reactivations (OR 3.914, *p* = 0.008); in contrast, both single and combined HHV reactivations did not show themselves to be independent predictors of overall mortality.

## 4. Discussion

HHVs occur at a high prevalence in the human population and are associated with a wide spectrum of clinical manifestations, ranging from asymptomatic infection to severe disease. They are characterized by their ability to establish life-long latent infections that can be interrupted by periods of intermittent lytic reactivation [23,24]. Amongst the known various environmental factors that can upset this delicate balance, a relevant role is played by various co-infections [25]. In this context, SARS-CoV-2 makes no exception, but many aspects remain to be clarified [26].

Based on these premises, this research systematically investigated the reactivation of all known HHVs in a group of patients with SARS-CoV-2 infection stratified into homogeneous sub-categories in terms of disease severity and compared to a control group not affected by COVID-19 [27]. In this regard, the two groups were generally similar in terms of clinical and demographic characteristics, although a potential impact on the obtained results could come from the fact that SARS-CoV-2-negative subjects unexpectedly had lower rates of known COPD, which is instead a high-risk underlying condition for COVID-19 (and, with a lesser degree of evidence, also for HHV reactivations). These considerations aside, the most relevant result that emerged is that an increased level of reactivation could be demonstrated for many HHVs in the SARS-CoV-2 infected cohort in comparison to the control group. Specifically, this was true for HSV-1, EBV, and HHV-6. Instead, no positives amongst SARS-CoV-2-infected subjects emerged concerning HSV-2, VZV, and HHV-8, but it should be borne in mind that most of these are notoriously hardly detectable (in their active forms with detectable viremia), even in healthy persons [28,29,30]. This is because, as mentioned earlier, they remain for most of host lives in latent forms [11,31,32]. Moreover, all of them were also poorly or not at all represented in the SARS-CoV-2-negative group. From a very general point of view, this phenomenon can easily be interpreted in the light of the known complex immune alterations caused by SARS-CoV-2, which in any case facilitates the reactivation of HHV regardless of any other known factors. As a matter of fact, existing SARS-CoV-2 infection (but probably also COVID-19 treatments) may promote viral reactivations and coinfections by impairing cellular immunity (with significant loss of lymphocytes or natural killer (NK) cells, and reduction or exhaustion of functional T cells with altered CD4+/CD8+ ratio). But other mechanisms probably also play a role. SARS-CoV-2-induced elevated levels of pro-inflammatory cytokines (such as IL-6, tumor necrosis factor-alpha and interferon-gamma) can create an environment conducive to HHV reactivation, modulating the expression of viral genes and promoting the lytic cycle. Additionally, the viral proteins of SARS-CoV-2 could interact with cellular pathways that regulate viral latency. All these aspects have been variously discussed in the recent literature and, although there is accumulating evidence, specific pathophysiological mechanisms of COVID-19 and HHV reactivations and coinfections are yet to be established [26,31,33,34,35,36,37,38,39].

If anything, what is less agreed upon between the various authors is the exact role of the severity degrees of COVID-19 manifestations in determining such concomitant increased reactivation of HHV. Therefore, it is no coincidence that this was one of the main objects of investigation in the current research. What clearly emerged from our results was that an incremental increase in the prevalence of HHV reactivation could be demonstrated the more severe the overall disease was. This included not only the previously cited viruses when dealing with the whole cohort of SARS-CoV-2-positive subjects (HSV-1; HHV-6; and, to a lesser extent, EBV), but also HCMV and HHV-7. Moreover, the same phenomenon was consequently more evident in those COVID-19 patients admitted to ICUs. This finding is in accordance with previously reported viral reactivation rates of up to over 50% in this subset of individuals [40,41,42,43,44,45]. Explaining this effect, at least in part, both aforementioned severe COVID-induced lymphopenia and mechanical ventilation are known risk factors for any secondary viral infections. To note, the reactivation of these opportunistic viral infections was described as a common event in ICU patients also with no previous immune suppression, which is precisely what was observed in our study population [38,46].

Specifically, HCMV was the only HHV as a single reactivation that confirmed its specific deleterious role, even when focusing on hospitalized subjects. Moreover, the same virus was also more prevalent in those individuals requiring longer durations of hospitalization, confirming consolidate evidence that HCMV coinfection is common in the severely ill [47]. In addition, HMCV was demonstrated to be an independent predictor of many relevant clinical adverse outcomes. To the best of our knowledge, these reports on a possible additive effect of HMCV on SARS-CoV-2 burden are rather unreported in the literature and, in any case, are non-univocal. This is because the full impact of this coinfection on the prognosis of COVID-19 remains largely unknown and, as a result, is underestimated by most treating physicians, despite the recent linkage of severe COVID-19 to substantial innate immune suppression, which may trigger HCMV [48]. Additionally, HCMV may further exacerbate COVID-related immune dysfunction in more fragile individuals, as it is able to target the immune system too [49,50]. Also, the real epidemiological impact of HCMV coinfections remains not completely understood. In some reports, this reactivation was the most detected concomitant infection [37], but other authors found lower prevalences outside the ICU setting [51,52,53]. In any case, most of the scarcely available evidence is in favor of the fact that HCMV reactivation is usually linked with poor clinical outcomes [33,45,52,54,55].

As previously discussed, besides HMCV, other viruses demonstrated some clinical interest in our analysis. Specifically, this was particularly evident for HSV-1, HHV-6, and HHV-7. As a matter of fact, all HHV expect EBV were more frequently activated in the symptomatic forms of COVID-19, explaining why they were generally associated with longer median hospitalizations and lymphocytopenia. Moreover, HSV-1 and HHV-7 were significantly more represented in ICU-admitted patients, as reported above. Anyway, all failed to act as independent predictors for any of the analyzed hard endpoints.

These last findings are partially in disagreement with earlier reports that, in turn, suggested that one or more of these viruses might indeed have a strong prognostic significance. In particular, such considerations concern the HSV-1 and EBV roles, which were most emphasized during the first pandemic waves of COVID-19. As a matter of fact, at least for what concerns these latter two viruses, systemic or pulmonary reactivations have frequently (as high as > 40%) been reported in the subset of individuals with severe COVID-19, posing a not-yet-solved dilemma for clinicians in terms of their diagnostic and clinical relevance. In our opinion, the biggest issue consists in the large heterogeneity across the various available studies, likely reflecting huge differences in study design; sample size; outcome measure; type of biological sample; diagnostic methods; and/or, last but not the least, cut-offs for defining reactivation [33,35,38,56,57,58,59,60,61]. From a more general perspective, there is frequently agreement on the clinical significance of these reactivations in the presence of severe manifestations clearly attributable to single viruses. However, the clinical implications of these coinfections and reactivations in the absence of manifest signs and symptoms remain controversial. Moreover, it is occasionally difficult to directly link respiratory symptoms to the SARS-CoV-2 infection or to the specific virus(es) involved in each single case.

All the considerations made so far apply to single HHV reactivations. When considering multiple (i.e., >1) reactivations, this evidence was not only confirmed but, indeed, reinforced. First of all, they appeared to be more represented in SARS-CoV-2-positive subjects, and with a strong linear trend as the severity of COVID-19 increased. Moreover, the same phenomena were elicited as the number of concomitant reactivations rose. Even more importantly, multiple (≥3) reactivations were more represented in the subjects suffering from the most severe forms of COVID-19. Moreover, they proved to be independent predictors of some clinically relevant hard endpoints, such as the occurrence of severe/critical COVID-19 forms or the need for long (>14 days) hospital stays. However—in analogy to the single reactivations—they did not demonstrate to be independent predictors of overall survival.

It is the authors’ opinion that these results may constitute one of the most innovative aspects of this research. There is a strong rationale for such an occurrence from a pathophysiological point of view, as it is well known that in COVID-19 pathology the most severe clinical situations generally involve immunocompromised individuals or with other chronic diseases, as confirmed also in our case series [62,63,64]. In these subjects, HHV reactivation may already be frequent but is most likely favored by the simultaneous SARS-CoV-2 infection [65,66,67]. We also know, however, that viral reactivation in these subjects can cause severe lung disease on its own. The previously mentioned fact of the simultaneous presence of SARS-CoV-2 and reactivated HHV during severe disease may therefore indicate a synergy between all these viruses in inducing pulmonary damage.

All these aspects taken into consideration, it must however be said that—to the best of our knowledge—the specific evidence accumulated to date is still rather scanty. Not considering reports on the muco-cutaneous systemic manifestations of HHVs, most authors reported that the reactivation of multiple HHVs at a time can increase the disease severity, at least in critically ill COVID-19 patients [43,44,63,64,68,69,70,71,72]. Moreover, some researchers described that multiple reactivations (mainly HSV-1, EBV, and HCMV) may have an association with the dosage and duration of corticosteroid usage (which was confirmed also by us) [43,69]. In any case, no study explicitly tested multiple reactivations as a primary aim, and cumulative reported positives were generally two or three, almost never exceeding four.

From all these considerations, we therefore believe that it would be crucial to monitor HHV reactivations in SARS-CoV-2 moderate-to-severe infections. Few antiviral drugs are active against SARS-CoV-2 and have limited antiviral activity. For some HHVs, on the other hand, we have drugs with excellent antiviral activity that allow potential control of reactivations, particularly for what concerns HSV-1 and HCMV. Incidentally, these two viruses in our study appeared to be the most implicated in the deleterious synergy with SARS-CoV-2. Obviously, it would be necessary to refine what the exact predictors of poor prognosis are, so as to improve the cost-effectiveness of such a strategy, beyond a generic recommendation to treat the most urgent cases. In this respect, future research of HHV and SARS-CoV-2 coinfections should be prioritized in order to define who, when, and how to be tested [37,39,59,73,74].

As a final remark, this study also has several limitations that should be considered when interpreting the results. First of all, the research was conducted during the fifth pandemic wave of COVID-19 in Italy, so it would be interesting to see if the same conclusions could be reached with the current SARS-CoV-2 variants, at least for the very small subset of patients who still experience a severe disease. Secondly, the relatively small sample size may lead to an insufficient statistical power for subgroup analyses, and the reported differences in the prevalence of underlying lung disease in the SARS-CoV-2-negative group with acute respiratory failure could also condition a statistical bias. Also, being a retrospective study, data are lacking on specific dermatological or neurological symptoms attributable to HHV coinfections. Moreover, as we verified the presence of HHV only in nasopharyngeal swabs, not exploring other tissues or materials, it would be useful to extend this surveillance also in other districts of the body (e.g., peripheral blood, stools, urine, lower respiratory tract material, etc.). Finally, this study did not analyze some aforementioned important immune mechanisms, such as lymphocyte subsets and NK cell activity, which should be included in future prospective research.

## 5. Conclusions

In this study, the prevalence of HHV reactivation in SARS-CoV-2-positive patients was significantly higher compared to negative subjects; these data confirmed that SARS-CoV-2 favors their reactivation. When focusing on the more severe COVID-19 infections, there seemed to be an increased HCMV reactivation rate, which could unfavorably affect the prognosis. These phenomena were elicited when multiple HHV reactivations were co-existing in the same subjects.

Taken together, our data suggest that, in patients with symptomatic COVID-19, the diagnosis of HHV coinfections could have a clinical utility, in that it would help to predict a possible worsening of the clinical conditions.

## Figures and Tables

**Table 1:** Main baseline clinical and demographic characteristics of the study population (*n* = 153). Data are presented as medians (interquartile range) for continuous variables and as frequencies (%) for categorical variables. Bold values denote statistical significance at the *p* < 0.05 level.

Variable	All Subjects (*n* = 153)	SARS-CoV-2-Positive Subjects (*n* = 102)	SARS-CoV-2-Negative Subjects (*n* = 51)	*p*
Male/female, *n*	98 (64)/55 (36)	66 (65)/36 (35)	32 (62)/19 (38)	0.952
Age, years	62.2 (50.3–71.4)	62.6 (54.4–71.9)	58.0 (39.9–69.5)	0.068
Disease severity				
asymptomatic/mild, *n*	34 (22)	34 (33)	-	
moderate, *n*	67 (44)	32 (32)	35 (69)	**0.025** ^1^
severe/critical, *n*	52 (34)	36 (35)	16 (31)	
Comorbidities ^2^, *n*				
Cardiovascular diseases ^3^, *n*	54 (35)	32 (31)	22 (43)	0.209
Diabetes mellitus, *n*	38 (25)	28 (27)	10 (20)	0.389
COPD, *n*	29 (19)	11 (11)	18 (35)	**<0.001**
Chronic kidney disease, *n*	23 (15)	18 (18)	5 (10)	0.298
Liver cirrhosis, *n*	11 (7)	7 (7)	4 (8)	0.911
Active malignancy ^4^, *n*	21 (14)	15 (15)	6 (12)	0.803
≥2 comorbidities, *n*	98 (64)	62 (61)	36 (71)	0.311
Immunosuppressive therapy ^5^, *n*	12 (8)	7 (7)	5 (10)	0.749
COVID-19 vaccination coverage ^6^, *n*	90 (59)	52 (51)	38 (75)	**0.006**
Main laboratory findings				
Lymphocyte count, ×10^3^/µL	995 (680–1650)	0.850 (0.615–1.180)	1.650 (1.310–2.210)	**<0.001**
Platelets, ×10^9^/L	197 (146–263)	185 (131–243)	214 (157–268)	0.226
C-reactive protein, mg/dL	6.0 (3.0–12.0)	6.2 (3.0–12.7)	9.0 (4.5–11.5)	0.779
D-dimer, ng/mL	1302 (593–2808)	1311 (710–3629)	593 (556–1302)	0.092
Troponin I, ng/L	9.0 (4.0–29.0)	10.5 (3.0–31.0)	8.0 (5.0–19.0)	0.667
Fibrinogen, mg/dL	468 (365–576)	480 (412–600)	295 (255–428)	**0.027**
LDH, U/L	507 (363–622)	530 (382–625)	380 (319–431)	0.043
Ferritin, ng/mL	583 (194–1009)	676 (217–1066)	172 (86–302)	**0.019**
AST, U/L	25 (19–41)	28 (19–48)	24 (20–28)	0.496
ALT, U/L	26 (17–38)	27 (19–39)	18 (17–30)	0.105
Interleukin-6, pg/mL ^7^	-	30.0 (15.2–39.2)	-	-
Total bilirubin, mg/dL	0.70 (0.53–0.90)	0.70 (0.50–0.90)	0.82 (0.67–1.22)	0.138
Creatinine, mg/dL	0.88 (0.67–1.18)	0.87 (0.63–1.37)	0.92 (0.72–1.06)	0.696
SOFA score ^1,8^ [19,20]				
0–3 ^9^, *n*	62 (52)	39 (57)	23 (45)	0.310
4–8 ^10^, *n*	47 (40)	25 (37)	22 (44)	
≥9 ^11^, *n*	10 (8)	4 (6)	6 (11)	
GCS ^1,8^, score	15 (15–15)	15 (15–15)	15 (12–15)	0.406
PaO_2_/FiO_2_, ratio ^1,8^	211 (127–284)	221 (124–317)	210 (145–270)	0.872
Respiratory rate ^1,8^, breaths per min	16 (16–26)	16 (16–25)	18 (16–29)	0.254
Length of hospital stay ^1^, days	12.1 (8.8–22.0)	19.0 (8.5–37.0)	9.4 (8.7–16.7)	**0.010**
Outcome ^12^				
Discharge, *n*	127 (83)	79 (77)	48 (94)	**0.009**
Death, *n*	26 (17)	23 (23)	3 (6)	

Abbreviations: aspartate aminotransferase (AST); alanine aminotransferase (ALT); chronic obstructive pulmonary disease (COPD); coronavirus disease 2019 (COVID-19); fraction of inspired oxygen (FiO_2_); Glasgow coma scale, (GCS); lactate dehydrogenase (LDH); arterial partial pressure of oxygen (PaO_2_); severe acute respiratory syndrome coronavirus 2 (SARS-CoV-2); sequential organ failure assessment (SOFA); ^1^ considering only 119 hospitalized patients (i.e., with moderate or severe/critical illness); ^2^ patients could have more than one comorbidity; ^3^ including systemic arterial hypertension; ^4^ including both solid tumors and hematological malignancies; ^5^ intended as a chronic therapy for at least two consecutive weeks prior to admission and any cause unrelated to SARS-CoV-2; ^6^ intended as a positive vaccination history with at least 3 doses at the time of admission (or at least 2 doses in subjects who were initially given a single-dose vaccine); ^7^ not routinely tested in our hospital for SARS-CoV-2-negative subjects; ^8^ at the first detection of clinical or laboratory parameters after the emergence of SARS-CoV-2-positivity (or at the first available determination for COVID-19-negative patients); ^9^ no organ failures; ^10^ mild organ(s) dysfunction; ^11^ moderate to severe organ(s) dysfunction; ^12^ within 60 days from the first SARS-CoV-2 positivity or from the date of hospitalization in SARS-CoV-2 negative individuals.

**Table 2:** Additional clinical and laboratory parameters of the group of SARS-CoV-2-positive patients (*n* = 102). Data are presented as medians (interquartile range) for continuous variables and as frequencies (%) for categorical variables.

Variable	
SARS-CoV-2 viral load	
Time interval for sample detection ^1^, days	1.8 (0.8–3.5)
PCR Ct values ^2^, *n*	28 (22–33)
Novara-COVID score [21]	
≤3, *n*	66 (65)
>3, *n*	36 (35)
Respiratory variables	
COVID-19 pneumonia ^3^, *n*	42 (41)
Pulmonary impairment ^4^	
<10%, *n*	51 (50)
10–50%, *n*	40 (39)
≥50%, *n*	11 (11)
Supplemental oxygen therapy ^5^	
None, *n*	36 (35)
Nasal cannula/Venturi mask, *n*	23 (23)
HFNC/CPAP/NIV, *n*	25 (24)
Endotracheal intubation, *n*	18 (18)
Length of ICU stay, days	23 (16–41)
Systemic corticosteroid treatment ^6,7^, *n*	70 (69)
Remdesivir treatment ^6^, *n*	100 (98)

Abbreviations: coronavirus disease (COVID); continuous positive airway pressure (CPAP); cycle threshold (Ct); high flow nasal cannula (HFNC); intensive care unit (ICU); non-invasive ventilation (NIV); polymerase chain reaction (PCR); severe acute respiratory syndrome coronavirus 2 (SARS-CoV-2); ^1^ time interval from the first reported symptoms and the collection of nasopharyngeal swabs in the subset of symptomatic SARS-CoV-2-positive subjects (*n* = 74); ^2^ at first molecular detection of SARS-CoV-2; ^3^ main reported diagnosis at hospital admission ^4^ at chest X-ray or computed tomography of the chest; ^5^ maximum level of support reached during hospital stay; ^6^ for COVID-19 treatment; ^7^ endovenous dexamethasone at the dosage of 6 mg/daily for 5–10 days [6,22].

**Table 3:** Analysis of herpesvirus reactivation(s) of SARS-CoV-2-negative and -positive subjects in the study population. Data are presented as frequencies (%). Bold values denote statistical significance at the *p* < 0.05 level. (**a**) Comparison of observed prevalences of herpesvirus reactivation(s) between SARS-CoV-2-negative and -positive subjects; (**b**) χ^2^ test for linear trend of herpesvirus reactivation(s) among SARS-CoV-2-negative and -positive subjects with asymptomatic-to-mild, moderate, and severe-to-critical disease; (**c**) χ^2^ test for linear trend of herpesvirus reactivation among SARS-CoV-2-positive subjects in the three considered disease stages.

Herpesvirus Reactivations	All Subjects (*n* = 153)	SARS-CoV-2- Negative Subjects (*n* = 51)	SARS-CoV-2-Positive Subjects				(a)	(b)	(c)
			Mild (*n* = 34)	Moderate (*n* = 32)	Severe (*n* = 36)	All (*n* = 102)	*p*		
HSV-1, *n*	28 (18)	1 (2)	3 (9)	8 (25)	16 (44)	27 (27)	**<0.001**	**<0.001**	**0.008**
HSV-2, *n*	0 (0)	0 (0)	0 (0)	0 (0)	0 (0)	0 (0)	-	-	-
VZV, *n*	0 (0)	0 (0)	0 (0)	0 (0)	0 (0)	0 (0)	-	-	-
HCMV, *n*	10 (7)	2 (4)	0 (0)	0 (0)	8 (22)	8 (8)	0.497	**0.015**	**0.012**
EBV, *n*	49 (32)	9 (18)	9 (26)	14 (44)	17 (47)	40 (39)	**0.009**	**0.002**	0.102
HHV-6, *n*	58 (38)	11 (22)	7 (21)	20 (62)	20 (56)	47 (46)	**0.004**	**<0.001**	**0.040**
HHV-7, *n*	78 (51)	20 (39)	14 (41)	18 (56)	26 (72)	58 (57)	0.059	**0.003**	**0.027**
HHV-8, *n*	1 (1)	1 (2)	0 (0)	0 (0)	0 (0)	0 (0)	0.333	0.114	-
≥1 reactivation, *n*	116 (76)	31 (61)	22 (65)	29 (91)	34 (94)	85 (83)	**0.005**	**<0.001**	**0.002**
≥2 reactivations, *n*	71 (46)	14 (27)	8 (24)	21 (66)	28 (78)	57 (56)	**0.001**	**<0.001**	**<0.001**
≥3 reactivations, *n*	27 (18) ^1^	0 (0)	2 (6)	8 (25)	17 (47)	27 (26)	**<0.001**	**<0.001**	**<0.001**

Abbreviations: Epstein–Barr virus (EBV); human cytomegalovirus (HCMV); human herpesvirus (HHV); herpes simplex virus (HSV); varicella-zoster virus (VZV); ^1^ of whom were 19 subjects with 3 HHV coinfections, 7 with 4 HHV coinfections, and 1 with all 5 HHV coinfections.

**Table 4:** Comparison of main clinical and demographic characteristics of all SARS-CoV-2-positive individuals (*n* = 102) with and without HHV reactivations. (**a**) HSV-1, HCMV, and EBV reactivations; (**b**) HHV-6, HHV-7, and ≥3 any HHV reactivations. Data are presented as medians (interquartile range) for continuous variables and as frequencies (%) for categorical variables. Bold values denote statistical significance at the *p* < 0.05 level.

(a)									
	HSV-1 Reactivation			HCMV Reactivation			EBV Reactivation		
	Yes	No	*p*	Yes	No	*p*	Yes	No	*p*
	(*n* = 27)	(*n* = 75)		(*n* = 8)	(*n* = 94)		(*n* = 40)	(*n* = 62)	
Ct value ≤ 20 of SARS-CoV-2 PCR, *n*	4 (15)	18 (24)	0.418	0 (0)	22 (23)	0.196	8 (30)	14 (19)	0.278
Male sex, *n*	14 (52)	52 (69)	0.158	4 (50)	62 (66)	0.448	28 (70)	38 (61)	0.403
Age, years	67.1 (60.7–75.8)	61.5 (53.5–70.1)	0.124	71.1 (55.1–81.2)	62.3 (53.9–72.6)	0.289	65.9 (54.4–71.4)	61.5 (55.2–73.6)	0.818
≥1 comorbidity, *n*	20 (74)	43 (57)	0.167	4 (50)	59 (63)	0.477	25 (62)	38 (61)	1.000
Lymphocyte count <1000 × 10^3^/µL, *n*	6 (22)	28 (37)	0.233	1 (12)	33 (35)	0.263	18 (45)	16 (26)	0.054
CRP > 4.0 mg/dL, *n*	18 (67)	42 (56)	0.370	8 (100)	52 (55)	0.020	25 (62)	35 (56)	0.681
D-dimer > 1000 ng/mL, *n*	20 (74)	42 (56)	0.113	6 (75)	56 (60)	0.476	24 (60)	38 (61)	1.000
IL-6 > 30 pg/mL, *n*	20 (74)	21 (28)	**<0.001**	6 (75)	35 (37)	0.058	22 (55)	19 (31)	**0.022**
Symptomatic disease ≥ mild, *n*	25 (93)	49 (65)	**0.006**	8 (100)	66 (70)	0.103	33 (82)	41 (66)	0.110
Corticosteroid use, *n*	22 (81)	48 (64)	0.146	5 (62)	65 (69)	0.703	25 (62)	45 (73)	0.382
Impairment of lung ≥ 10%, *n*	16 (59)	35 (47)	0.370	7 (88)	44 (47)	0.060	22 (55)	29 (47)	0.543
Respiratory treatment need ^1^, *n*	15 (56)	28 (37)	0.116	6 (75)	37 (39)	0.067	21 (52)	22 (35)	0.103
ICU admission, *n*	15 (56)	21 (28)	**0.018**	6 (75)	30 (32)	**0.022**	17 (42)	19 (31)	0.289
Hospital stay, days	22.5 (8.5–38.5)	8.0 (0.0–19.0)	**0.002**	42.0 (37.5–53.5)	9.1 (0.0–21.8)	**<0.001**	13.0 (5.5–29.5)	9.0 (0.0–25.9)	0.379
Death ^2^, *n*	10 (37)	13 (17)	0.058	2 (25)	21 (22)	1.000	15 (37)	8 (13)	**0.007**
(**b**)									
	**HHV-6 reactivation**		***p***	**HHV-7 reactivation**		***p***	**≥3 any reactivations**		***p***
	**yes**	**no**		**yes**	**no**		**yes**	**no**	
	**(*n* = 47)**	**(*n* = 55)**		**(*n* = 58)**	**(*n* = 44)**		**(*n* = 27)**	**(*n* = 75)**	
Ct value ≤ 20 of SARS-CoV-2 PCR, *n*	7 (15)	15 (27)	0.153	12 (21)	10 (23)	0.813	7 (26)	15 (20)	0.588
Male sex, *n*	33 (70)	33 (60)	0.306	39 (67)	27 (61)	0.676	18 (67)	48 (64)	1.000
Age, years	62.4 (55.5–72.1)	62.7 (53.9–72.3)	0.802	66.6 (56.9–75.1)	60.2 (53.8–68.1)	0.105	67.1 (57.9–75.6)	61.5 (53.3–70.4)	0.162
≥1 comorbidity, *n*	26 (55)	37 (67)	0.228	37 (64)	26 (59)	0.683	18 (67)	45 (60)	0.646
Lymphocyte count <1000 × 10^3^/µL, *n*	9 (19)	25 (45)	**0.006**	25 (43)	9 (20)	**0.020**	8 (30)	26 (35)	0.812
CRP > 4.0 mg/dL, *n*	34 (72)	26 (47)	**0.015**	39 (67)	21 (48)	0.067	23 (85)	37 (49)	**0.001**
D-dimer > 1000 ng/mL, *n*	27 (57)	35 (64)	0.548	39 (67)	23 (52)	0.153	19 (70)	43 (57)	0.259
IL-6 > 30 pg/mL, *n*	20 (43)	21 (38)	0.689	25 (43)	16 (36)	0.545	15 (56)	26 (35)	0.069
Symptomatic disease ≥ mild, *n*	42 (89)	32 (58)	**<0.001**	48 (83)	26 (59)	**0.013**	25 (93)	49 (65)	**0.006**
Corticosteroid use, *n*	36 (77)	34 (62)	0.136	46 (79)	24 (55)	**0.010**	25 (93)	45 (60)	**0.001**
Impairment of lung ≥ 10%, *n*	28 (60)	23 (42)	0.112	36 (62)	15 (34)	**0.009**	18 (67)	33 (44)	0.071
Respiratory treatment need ^1^, *n*	24 (51)	19 (35)	0.110	26 (45)	17 (39)	0.551	16 (59)	27 (36)	**0.042**
ICU admission, *n*	20 (43)	16 (29)	0.212	25 (43)	11 (25)	0.064	16 (59)	20 (27)	**0.004**
Hospital stay, days	17.5 (7.3–37.8)	7.2 (0.0–21.4)	**0.009**	17.1 (7.2–30.3)	6.2 (0.0–18.4)	**0.015**	26.1 (8.5–41.2)	7.3 (0.0–19.4)	**<0.001**
Death ^2^, *n*	12 (26)	11 (20)	0.635	17 (29)	6 (14)	0.093	9 (33)	14 (19)	0.128

Abbreviations: continuous positive airway pressure (CPAP); C-reactive protein (CRP); Epstein–Barr virus (EBV); endotracheal intubation (ETI); human cytomegalovirus (HCMV); high flow nasal cannula (HFNC); herpes simplex virus (HSV); intensive care unit (ICU); interleukin (IL); non-invasive ventilation (NIV); sequential organ failure assessment (SOFA); ^1^ HFNC, CPAP, NIV, or ETI; ^2^ within 60 days from the first SARS-CoV-2 positivity.

**Table 5:** Factors associated with unfavorable clinical outcomes in patients hospitalized for COVID-19 (*n* = 68). (**a**) Univariate analysis; (**b**) multivariate analysis. Bold values denote statistical significance at the *p* < 0.05 level.

	Occurrence of Severe/Critical Forms of COVID-19			Length of Hospital Stay > 14 Days			Non-Invasive ^1^ or Invasive ^2^ Ventilatory Assistance Need			Death Within 60 Days from First SARS-CoV-2 Positivity		
Factors	OR	95% CI	*p*	OR	95% CI	*p*	OR	95% CI	*p*	OR	95% CI	*p*
**(a)**												
Age (years)	0.985	0.942– 1.030	0.519	1.011	0.968– 1.057	0.599	1.027	0.982– 1.073	0.243	0.997	0.952– 1.043	0.898
Sex (male/female)	1.200	0.339– 4.243	0.777	1.784	0.717– 3.715	0.698	0.985	0.288– 3.372	0.981	1.296	0.358– 4.687	0.692
≥2 comorbidities	0.866	0.285– 2.632	0.800	1.764	0.563– 5.531	0.329	1.544	0.507– 4.695	0.443	1.026	0.319– 3.300	0.964
Lymphocyte count < 1000 × 10^3^/µL	1.000	0.313– 3.191	1.000	1.916	0.602– 6.101	0.270	0.558	0.172– 1.815	0.333	0.604	0.182– 1.998	0.409
IL-6 > 30 pg/mL	1.441	0.944– 4.851	0.997	1.285	0.158– 10.450	0.814	3.600	0.490– 26.398	0.207	18.666	1.563– 222.926	**0.020**
COVID-19 severity	-	-	-	13.500	3.520– 51.774	**<0.001**	11.885	3.220– 43.862	**<0.001**	2.294	1.137– 9.377	**0.015**
SOFA ≥ 4	1.354	1.443– 4.133	**0.034**	4.123	2.029– 21.358	**0.009**	4.028	2.579– 5.334	**0.021**	3.819	1.139– 12.803	**0.029**
Length of hospital stay > 14 days	-	-	**-**	-	-	**-**	-	-	**-**	1.920	0.559– 6.589	0.299
Corticosteroid use	2.784	0.384– 2.369	0.998	1.550	0.091– 26.219	0.761	1.378	0.518– 2.473	0.998	1.129	0.796– 4.713	0.997
Impairment of lung ≥ 10%	4.950	1.445– 16.955	**0.010**	1.846	1.561– 6.068	**0.019**	2.637	1.811– 8.568	**0.006**	12.190	1.459– 101.804	**0.020**
Ventilatory assistance need ^1,2^	11.885	3.220– 43.862	**<0.001**	6.000	1.789– 20.115	**0.003**	**-**	**-**	**-**	5.490	1.347– 22.366	**0.017**
HSV-1 reactivation	2.666	0.790– 8.993	0.113	1.944	0.599– 6.305	0.267	0.855	0.280– 2.608	0.783	1.851	0.571– 5.997	0.304
HCMV reactivation	2.987	1.397– 7.234	**0.026**	3.179	1.172– 4.765	**0.012**	2.019	0.955– 11.478	0.071	0.853	0.148– 4.914	0.859
EBV reactivation	1.254	0.417– 3.775	0.686	0.707	0.234– 2.134	0.538	1.218	0.412– 3.604	0.720	3.011	0.910– 9.961	0.070
HHV-6 reactivation	0.738	0.241– 2.254	0.594	1.328	0.438– 4.029	0.615	1.065	0.358– 3.168	0.909	0.767	0.241– 2.438	0.653
HHV-7 reactivation	2.000	0.630– 6.347	0.239	2.250	0.692– 7.306	0.177	2.211	0.698– 6.997	0.176	1.978	0.537– 7.279	0.304
Number of HHV reactivations	1.580	0.940– 2.656	0.083	1.533	0.914– 2.573	0.105	1.241	0.767– 2.007	0.378	1.454	0.858– 2.465	0.163
≥2 any HHV reactivations	1.562	0.467– 5.224	0.468	1.785	1.519– 13.140	**0.038**	1.828	0.549– 6.084	0.325	2.240	0.539– 9.307	0.267
≥3 any HHV reactivations	3.011	1.894– 10.143	**0.035**	4.367	2.561– 16.445	**0.002**	2.882	1.104– 5.858	**0.044**	1.641	0.510– 5.273	0.405
(**b**)												
**Factors**	**OR**	**95% CI**	***p***	**OR**	**95% CI**	***p***	**OR**	**95% CI**	***p***	**OR**	**95% CI**	***p***
IL-6 > 30 pg/mL	-	-	**-**	-	-	**-**	-	-		10.228	0.467– 223.967	0.139
COVID-19 severity	-	-	**-**	8.265	1.357– 50.332	**0.021**	10.668	2.555– 44.528	**0.001**	3.174	1.458– 13.412	**0.029**
SOFA ≥ 4	2.471	1.665– 5.863	**0.025**	12.000	1.423– 101.187	**0.022**	1.801	0.475– 6.826	0.386	5.345	1.347– 18.700	**0.022**
Impairment of lung ≥ 10%	3.726	1.370– 17.542	**0.005**	1.342	1.228– 6.219	**0.014**	1.236	1.284– 5.364	**0.032**	13.335	1.087– 180.125	**0.031**
Ventilatory assistance need ^1,2^	11.334	2.636– 48.728	**0.001**	2.755	0.956– 7.940	0.060	**-**	**-**	**-**	3.083	0.764– 24.140	0.091
HCMV reactivation	3.576	1.678– 9.189	**0.026**	2.224	1.364– 6.121	**0.031**	**-**	**-**	**-**	**-**	**-**	**-**
≥2 any HHV reactivations	-	-	**-**	1.822	0.277– 11.968	0.532	**-**	**-**	**-**	**-**	**-**	**-**
≥3 any HHV reactivations	2.623	1.308– 8.549	**0.041**	3.567	2.119– 19.453	**0.046**	2.192	0.897– 4.776	0.079	**-**	**-**	**-**

Abbreviations: confidence interval (CI); continuous positive airway pressure (CPAP); Epstein–Barr virus (EBV); endotracheal intubation (ETI); human cytomegalovirus (HCMV); high flow nasal cannula (HFNC); human herpesvirus (HHV); herpes simplex virus (HSV); intensive care unit (ICU); interleukin (IL); non-invasive ventilation (NIV); odds ratio (OR); sequential organ failure assessment (SOFA); ^1^ HFNC, CPAP, or NIV; ^2^ ETI.

## Data Availability

The original contributions presented in this study are included in the article. Further inquiries can be directed to the corresponding author.

## Supplementary Materials

The following supporting information can be downloaded at https://www.mdpi.com/article/10.3390/microorganisms13061221/s1. Supplementary Table S1: Comparison of the prevalence of reactivation of herpesviruses in different subgroups of SARS-COV-2-negative and positive subjects in the study population (*n* = 153), with a focus on more severe disease stages. Supplementary Table S2: Comparison of the cycle threshold (Ct) values of SARS-CoV-2 RT-PCR in the subjects with and without HHV reactivation(s). Supplementary Figure S1: Chest radiological imaging of the single SARS-CoV-2-positive 71-year-old female patient with all 5 HHV reactivations detected at the same time.

## Abbreviations

The following abbreviations are used in this manuscript:

ALT	Alanine aminotransferase
ANOVA	Analysis of variance
AST	Aspartate aminotransferase
CI	Confidence interval
COPD	Chronic obstructive pulmonary disease
COVID-19	Coronavirus disease 2019
CPAP	Continuous positive airway pressure
CRP	C-reactive protein
Ct	Cycle threshold
ETI	Endotracheal intubation
GCS	Glasgow coma scale
EBV	Epstein–Barr virus
FiO_2_	Fraction of inspired oxygen
GGO	Ground glass opacities
HHV	Human herpesviruses
HCMV	Human cytomegalovirus
HFNC	High flow nasal cannula
HIV	Human immunodeficiency virus
HSV	Herpes simplex virus
ICU	Intensive care unit
IL	Interleukin
LDH	Lactate dehydrogenase
NIV	Non-invasive ventilation
NK	Natural killer
NKG2A	Natural killer group 2 member A
OR	Odds ratio
PaO_2_	Arterial partial pressure of oxygen
PCR	Polymerase chain reaction
Q-Q	Quantile–quantile
RT	Real-time reverse transcriptase
SARS-CoV-2	Severe acute respiratory syndrome coronavirus 2
SOFA	Sequential organ failure assessment
VZV	Varicella-zoster virus

## References

[1] Tian S., Hu N., Lou J., Chen K., Kang X., Xiang Z., Chen H., Wang D., Liu N., Liu D. (2020). Characteristics of COVID-19 Infection in Beijing. J. Infect..

[2] Zhang J., Litvinova M., Wang W., Wang Y., Deng X., Chen X., Li M., Zheng W., Yi L., Chen X. (2020). Evolving Epidemiology and Transmission Dynamics of Coronavirus Disease 2019 Outside Hubei Province, China: A Descriptive and Modelling Study. Lancet Infect. Dis..

[3] Li G., Fan Y., Lai Y., Han T., Li Z., Zhou P., Pan P., Wang W., Hu D., Liu X. (2020). Coronavirus Infections and Immune Responses. J. Med. Virol..

[4] Shereen M.A., Khan S., Kazmi A., Bashir N., Siddique R. (2020). COVID-19 Infection: Origin, Transmission, and Characteristics of Human Coronaviruses. J. Adv. Res..

[5] Shi Y., Wang Y., Shao C., Huang J., Gan J., Huang X., Bucci E., Piacentini M., Ippolito G., Melino G. (2020). COVID-19 Infection: The Perspectives on Immune Responses. Cell Death Differ..

[6] Boglione L., Crobu M.G., Pirisi M., Smirne C. (2024). Clinical Characteristics and Outcomes in Patients with Chronic HBV Infection and Hospitalized for COVID-19 Pneumonia: A Retrospective Cohort Study. Viruses.

[7] Shafiee A., Teymouri Athar M.M., Nassar M., Seighali N., Aminzade D., Fattahi P., Rahmannia M., Ahmadi Z. (2022). Comparison of COVID-19 Outcomes in Patients with Type 1 and Type 2 Diabetes: A Systematic Review and Meta-Analysis. Diabetes Metab. Syndr..

[8] Chen T., Song J., Liu H., Zheng H., Chen C. (2021). Positive Epstein–Barr Virus Detection in Coronavirus Disease 2019 (COVID-19) Patients. Sci. Rep..

[9] Moss P. (2020). “The Ancient and the New”: Is There an Interaction between Cytomegalovirus and SARS-CoV-2 Infection?. Immun. Ageing.

[10] Virgin H.W. (2014). The Virome in Mammalian Physiology and Disease. Cell.

[11] Sehrawat S., Kumar D., Rouse B.T. (2018). Herpesviruses: Harmonious Pathogens but Relevant Cofactors in Other Diseases?. Front. Cell Infect. Microbiol..

[12] Shafiee A., Shamsi S., Kohandel Gargari O., Beiky M., Allahkarami M.M., Miyanaji A.B., Aghajanian S., Mozhgani S.H. (2022). EBV Associated T- and NK-Cell Lymphoproliferative Diseases: A Comprehensive Overview of Clinical Manifestations and Novel Therapeutic Insights. Rev. Med. Virol..

[13] Riaz A. (2017). Recent Understanding of the Classification and Life Cycle of Herpesviruses—A Review. Sci. Lett. J..

[14] Mendonça F.T., Mendonça F.T. (2020). Immunosuppressed Patients and the Risk of COVID-19: A Narrative Review. Clin. Oncol. Res..

[15] Ong D.S.Y., Bonten M.J.M., Spitoni C., Lunel F.M.V., Frencken J.F., Horn J., Schultz M.J., van der Poll T., Klouwenberg P.M.C.K., Cremer O.L. (2017). Epidemiology of Multiple Herpes Viremia in Previously Immunocompetent Patients with Septic Shock. Clin. Infect. Dis..

[16] Malekifar P., Pakzad R., Shahbahrami R., Zandi M., Jafarpour A., Rezayat S.A., Akbarpour S., Shabestari A.N., Pakzad I., Hesari E. (2021). Viral Coinfection among COVID-19 Patient Groups: An Update Systematic Review and Meta-Analysis. Biomed. Res. Int..

[17] Aghbash P.S., Eslami N., Shirvaliloo M., Baghi H.B. (2021). Viral Coinfections in COVID-19. J. Med. Virol..

[18] Feng Y., Ling Y., Bai T., Xie Y., Huang J., Li J., Xiong W., Yang D., Chen R., Lu F. (2020). COVID-19 with Different Severities: A Multicenter Study of Clinical Features. Am. J. Respir. Crit. Care Med..

[19] Pölkki A., Pekkarinen P.T., Takala J., Selander T., Reinikainen M. (2022). Association of Sequential Organ Failure Assessment (SOFA) Components with Mortality. Acta Anaesthesiol. Scand..

[20] Nair R., Bhandary N.M., D’Souza A.D. (2016). Initial Sequential Organ Failure Assessment Score versus Simplified Acute Physiology Score to Analyze Multiple Organ Dysfunction in Infectious Diseases in Intensive Care Unit. Indian. J. Crit. Care Med..

[21] Gavelli F., Castello L.M., Bellan M., Azzolina D., Hayden E., Beltrame M., Galbiati A., Gardino C.A., Gastaldello M.L., Giolitti F. (2021). Clinical Stability and In-Hospital Mortality Prediction in COVID-19 Patients Presenting to the Emergency Department. Minerva Med..

[22] Horby P., Lim W., Emberson J., Mafham M., Bell J., Linsell L., Staplin N., Brightling C., Ustianowski A., RECOVERY Collaborative Group (2021). Dexamethasone in Hospitalized Patients with COVID-19. N. Engl. J. Med..

[23] Boehmer P.E., Nimonkar A.V. (2003). Herpes Virus Replication. IUBMB Life.

[24] Grinde B. (2013). Herpesviruses: Latency and Reactivation—Viral Strategies and Host Response. J. Oral. Microbiol..

[25] Reese T.A. (2016). Coinfections: Another Variable in the Herpesvirus Latency-Reactivation Dynamic. J. Virol..

[26] Chen J., Song J., Dai L., Post S.R., Qin Z. (2022). SARS-CoV-2 Infection and Lytic Reactivation of Herpesviruses: A Potential Threat in the Postpandemic Era?. J. Med. Virol..

[27] CDC (US Centers for Disease Control and Prevention) COVID-19 (Version for Healthcare Workers): Clinical Presentation. https://www.cdc.gov/covid/hcp/clinical-care/covid19-presentation.html.

[28] Alareeki A., Osman A.M.M., Khandakji M.N., Looker K.J., Harfouche M., Abu-Raddad L.J. (2023). Epidemiology of Herpes Simplex Virus Type 2 in Europe: Systematic Review, Meta-Analyses, and Meta-Regressions. Lancet Reg. Health Eur..

[29] Gershon A.A., Breuer J., Cohen J.I., Cohrs R.J., Gershon M.D., Gilden D., Grose C., Hambleton S., Kennedy P.G.E., Oxman M.N. (2015). Varicella Zoster Virus Infection. Nat. Rev. Dis. Primers.

[30] Edelman D.C. (2005). Human Herpesvirus 8—A Novel Human Pathogen. Virol. J..

[31] Chinna P., Bratl K., Lambarey H., Blumenthal M.J., Schäfer G. (2023). The Impact of Co-Infections for Human Gammaherpesvirus Infection and Associated Pathologies. Int. J. Mol. Sci..

[32] White D.W., Suzanne Beard R., Barton E.S. (2012). Immune Modulation during Latent Herpesvirus Infection. Immunol. Rev..

[33] Shafiee A., Teymouri Athar M.M., Amini M.J., Hajishah H., Siahvoshi S., Jalali M., Jahanbakhshi B., Mozhgani S.H. (2023). Reactivation of Herpesviruses during COVID-19: A Systematic Review and Meta-Analysis. Rev. Med. Virol..

[34] Navarro-Bielsa A., Gracia-Cazaña T., Aldea-Manrique B., Abadías-Granado I., Ballano A., Bernad I., Gilaberte Y. (2023). COVID-19 Infection and Vaccines: Potential Triggers of Herpesviridae Reactivation. An. Bras. Dermatol..

[35] Carneiro V.C.d.S., Alves-Leon S.V., Sarmento D.J.d.S., Coelho W.L.d.C.N.P., Moreira O.d.C., Salvio A.L., Ramos C.H.F., Filho C.H.F.R., Marques C.A.B., Gonçalves J.P.d.C. (2022). Herpesvirus and Neurological Manifestations in Patients with Severe Coronavirus Disease. Virol. J..

[36] Roncati L., Sweidan E., Tchawa C., Gianotti G., Di Massa G., Siciliano F., Paolini A. (2023). SARS-CoV-2 Induced Herpes Virus Reactivations and Related Implications in Oncohematology: When Lymphocytopenia Sets in and Immunosurveillance Drops Out. Microorganisms.

[37] Talukder S., Deb P., Parveen M., Zannat K.E., Bhuiyan A.H., Yeasmin M., Molla M.M.A., Saif-Ur-Rahman K.M. (2024). Clinical Features and Outcomes of COVID-19 Patients with Concomitant Herpesvirus Co-Infection or Reactivation: A Systematic Review. New Microbes New Infect..

[38] Vojdani A., Vojdani E., Saidara E., Maes M. (2023). Persistent SARS-CoV-2 Infection, EBV, HHV-6 and Other Factors May Contribute to Inflammation and Autoimmunity in Long COVID. Viruses.

[39] Gáspár Z., Szabó B.G., Ceglédi A., Lakatos B. (2024). Human Herpesvirus Reactivation and Its Potential Role in the Pathogenesis of Post-Acute Sequelae of SARS-CoV-2 Infection. Geroscience.

[40] Saura O., Chommeloux J., Levy D., Assouline B., Lefevre L., Luyt C.E. (2022). Updates in the Management of Respiratory Virus Infections in ICU Patients: Revisiting the Non-SARS-CoV-2 Pathogens. Expert. Rev. Anti Infect. Ther..

[41] Fumagalli J., Panigada M., Klompas M., Berra L. (2022). Ventilator-Associated Pneumonia among SARSCoV-2 Acute Respiratory Distress Syndrome Patients. Curr. Opin. Crit. Care.

[42] Conway Morris A., Smielewska A. (2023). Viral Infections in Critical Care: A Narrative Review. Anaesthesia.

[43] Le Balc’h P., Pinceaux K., Pronier C., Seguin P., Tadié J.M., Reizine F. (2020). Herpes Simplex Virus and Cytomegalovirus Reactivations among Severe COVID-19 Patients. Crit. Care.

[44] Simonnet A., Engelmann I., Moreau A.S., Garcia B., Six S., El Kalioubie A., Robriquet L., Hober D., Jourdain M. (2021). High Incidence of Epstein–Barr Virus, Cytomegalovirus, and Human-Herpes Virus-6 Reactivations in Critically Ill Patients with COVID-19. Infect. Dis. Now..

[45] Taherifard E., Movahed H., Kiani Salmi S., Taherifard A., Abdollahifard S., Taherifard E. (2022). Cytomegalovirus Coinfection in Patients with Severe Acute Respiratory Syndrome Coronavirus 2 Infection: A Systematic Review of Reported Cases. Infect Dis.

[46] D’Ardes D., Boccatonda A., Schiavone C., Santilli F., Guagnano M.T., Bucci M., Cipollone F. (2020). A Case of Coinfection with SARS-CoV-2 and Cytomegalovirus in the Era of COVID-19. Eur. J. Case Rep. Intern. Med..

[47] Nakase H., Herfarth H. (2016). Cytomegalovirus Colitis, Cytomegalovirus Hepatitis and Systemic Cytomegalovirus Infection: Common Features and Differences. Inflamm. Intest. Dis..

[48] Tian W., Zhang N., Jin R., Feng Y., Wang S., Gao S., Gao R., Wu G., Tian D., Tan W. (2020). Immune Suppression in the Early Stage of COVID-19 Disease. Nat. Commun..

[49] Antonioli L., Fornai M., Pellegrini C., Blandizzi C. (2020). NKG2A and COVID-19: Another Brick in the Wall. Cell Mol. Immunol..

[50] Djaoud Z., Riou R., Gavlovsky P.J., Mehlal S., Bressollette C., Gérard N., Gagne K., Charreau B., Retière C. (2016). Cytomegalovirus-Infected Primary Endothelial Cells Trigger NKG2C+ Natural Killer Cells. J. Innate Immun..

[51] Luyt C.E., Girardis M., Paixão P. (2024). Herpes Simplex Virus and Cytomegalovirus Lung Reactivations in Severe COVID-19 Patients: To Treat or Not to Treat? That Is (Still) the Question. Intensive Care Med..

[52] Gatto I., Biagioni E., Coloretti I., Farinelli C., Avoni C., Caciagli V., Busani S., Sarti M., Pecorari M., Gennari W. (2022). Cytomegalovirus Blood Reactivation in COVID-19 Critically Ill Patients: Risk Factors and Impact on Mortality. Intensive Care Med..

[53] Söderberg-Nauclér C. (2021). Does Reactivation of Cytomegalovirus Contribute to Severe COVID-19 Disease?. Immun. Ageing.

[54] Pérez-Granda M.J., Catalán P., Muñoz P., Aldámiz T., Barrios J.C., Ramírez C., García-Martínez R., Villalba M.V., Puente L., Bouza E. (2023). Cytomegalovirus Reactivation in Patients Diagnosed with Severe COVID-19: A Point Prevalence Study in a General Hospital. Rev. Esp. Quimioter..

[55] Schinas G., Moustaka V., Polyzou E., Almyroudi M.P., Dimopoulos G., Akinosoglou K. (2023). Targeting CMV Reactivation to Optimize Care for Critically Ill COVID-19 Patients: A Review on the Therapeutic Potential of Antiviral Treatment. Viruses.

[56] Giacobbe D.R., Di Bella S., Lovecchio A., Ball L., De Maria A., Vena A., Bruzzone B., Icardi G., Pelosi P., Luzzati R. (2022). Herpes Simplex Virus 1 (HSV-1) Reactivation in Critically Ill COVID-19 Patients: A Brief Narrative Review. Infect. Dis. Ther..

[57] Meyer A., Buetti N., Houhou-Fidouh N., Patrier J., Abdel-Nabey M., Jaquet P., Presente S., Girard T., Sayagh F., Ruckly S. (2021). HSV-1 Reactivation Is Associated with an Increased Risk of Mortality and Pneumonia in Critically Ill COVID-19 Patients. Crit. Care.

[58] Brooks B., Tancredi C., Song Y., Mogus A.T., Huang M.L.W., Zhu H., Phan T.L., Zhu H., Kadl A., Woodfolk J. (2022). Epstein–Barr Virus and Human Herpesvirus-6 Reactivation in Acute COVID-19 Patients. Viruses.

[59] Kim J.Y.H., Ragusa M., Tortosa F., Torres A., Gresh L., Méndez-Rico J.A., Alvarez-Moreno C.A., Lisboa T.C., Valderrama-Beltrán S.L., Aldighieri S. (2023). Viral Reactivations and Co-Infections in COVID-19 Patients: A Systematic Review. BMC Infect. Dis..

[60] Solomay T.V., Semenenko T.A., Filatov N.N., Vedunova S.L., Lavrov V.F., Smirnova D.I., Gracheva A.V., Faizuloev E.B. (2021). Reactivation of Epstein-Barr Virus (Herpesviridae: Lymphocryptovirus, HHV-4) Infection during COVID-19: Epidemiological Features. Vopr. Virusol..

[61] Manoharan S., Ying L.Y. (2023). Epstein Barr Virus Reactivation during COVID-19 Hospitalization Significantly Increased Mortality/Death in SARS-CoV-2(+)/EBV(+) than SARS-CoV-2(+)/EBV(-) Patients: A Comparative Meta-Analysis. Int. J. Clin. Pract..

[62] Busnadiego I., Abela I.A., Frey P.M., Hofmaenner D.A., Scheier T.C., Schuepbach R.A., Buehler P.K., Brugger S.D., Hale B.G. (2022). Critically Ill COVID-19 Patients with Neutralizing Autoantibodies against Type I Interferons Have Increased Risk of Herpesvirus Disease. PLoS Biol..

[63] Saade A., Moratelli G., Azoulay E., Darmon M. (2021). Herpesvirus Reactivation during Severe COVID-19 and High Rate of Immune Defect. Infect. Dis. Now..

[64] Vigón L., García-Pérez J., Rodríguez-Mora S., Torres M., Mateos E., Castillo de la Osa M., Cervero M., Malo De Molina R., Navarro C., Murciano-Antón M.A. (2021). Impaired Antibody-Dependent Cellular Cytotoxicity in a Spanish Cohort of Patients With COVID-19 Admitted to the ICU. Front. Immunol..

[65] Huang R.C., Chiu C.H., Chiang T.T., Tsai C.C., Wang Y.C., Chang F.Y., Yang Y.S., Wang C.H. (2022). Hospital-Acquired Infections in Patients Hospitalized with COVID-19: First Report from Taiwan. J. Chin. Med. Assoc..

[66] Paolucci S., Cassaniti I., Novazzi F., Fiorina L., Piralla A., Comolli G., Bruno R., Maserati R., Gulminetti R., Novati S. (2021). EBV DNA Increase in COVID-19 Patients with Impaired Lymphocyte Subpopulation Count. Int. J. Infect. Dis..

[67] Paparoupa M., Aldemyati R., Roggenkamp H., Berinson B., Nörz D., Olearo F., Kluge S., Roedl K., de Heer G., Wichmann D. (2022). The Prevalence of Early- and Late-Onset Bacterial, Viral, and Fungal Respiratory Superinfections in Invasively Ventilated COVID-19 Patients. J. Med. Virol..

[68] Textoris J., Mallet F. (2017). Immunosuppression and Herpes Viral Reactivation in Intensive Care Unit Patients: One Size Does Not Fit All. Crit. Care.

[69] Reizine F., Liard C., Pronier C., Thibault V., Maamar A., Gacouin A., Tadié J.M. (2022). Herpesviridae Systemic Reactivation in Patients with COVID-19-Associated ARDS. J. Hosp. Infect..

[70] Fuest K.E., Erber J., Berg-Johnson W., Heim M., Hoffmann D., Kapfer B., Kriescher S., Ulm B., Schmid R.M., Rasch S. (2022). Risk Factors for Herpes Simplex Virus (HSV) and Cytomegalovirus (CMV) Infections in Critically-Ill COVID-19 Patients. Multidiscip. Respir. Med..

[71] Blumenthal M.J., Lambarey H., Chetram A., Riou C., Wilkinson R.J., Schäfer G. (2022). Kaposi’s Sarcoma-Associated Herpesvirus, but Not Epstein-Barr Virus, Co-Infection Associates with Coronavirus Disease 2019 Severity and Outcome in South African Patients. Front. Microbiol..

[72] Mattei A., Schiavoni L., Riva E., Ciccozzi M., Veralli R., Urselli A., Citriniti V., Nenna A., Pascarella G., Costa F. (2024). Epstein–Barr Virus, Cytomegalovirus, and Herpes Simplex-1/2 Reactivations in Critically Ill Patients with COVID-19. Intensive Care Med. Exp..

[73] Banko A., Miljanovic D., Cirkovic A. (2023). Systematic Review with Meta-Analysis of Active Herpesvirus Infections in Patients with COVID-19: Old Players on the New Field. Int. J. Infect. Dis..

[74] Grubelnik G., Korva M., Kogoj R., Polanc T., Mavrič M., Jevšnik Virant M., Uršič T., Keše D., Seme K., Petrovec M. (2024). Herpesviridae and Atypical Bacteria Co-Detections in Lower Respiratory Tract Samples of SARS-CoV-2-Positive Patients Admitted to an Intensive Care Unit. Microorganisms.

