# Supplementary material for: Clinical Features and Outcomes of Patients with Full Spectrum of COVID-19 Severity and Concomitant Herpesvirus Reactivation
Source: Microorganisms. 2025 May 27;13(6):1221. doi: 10.3390/microorganisms13061221 (PMC12195333; doi:10.3390/microorganisms13061221)
Supplement: Supplementary file 1 [file microorganisms-13-01221-s001.zip › Supplementary Figure S1.pdf]

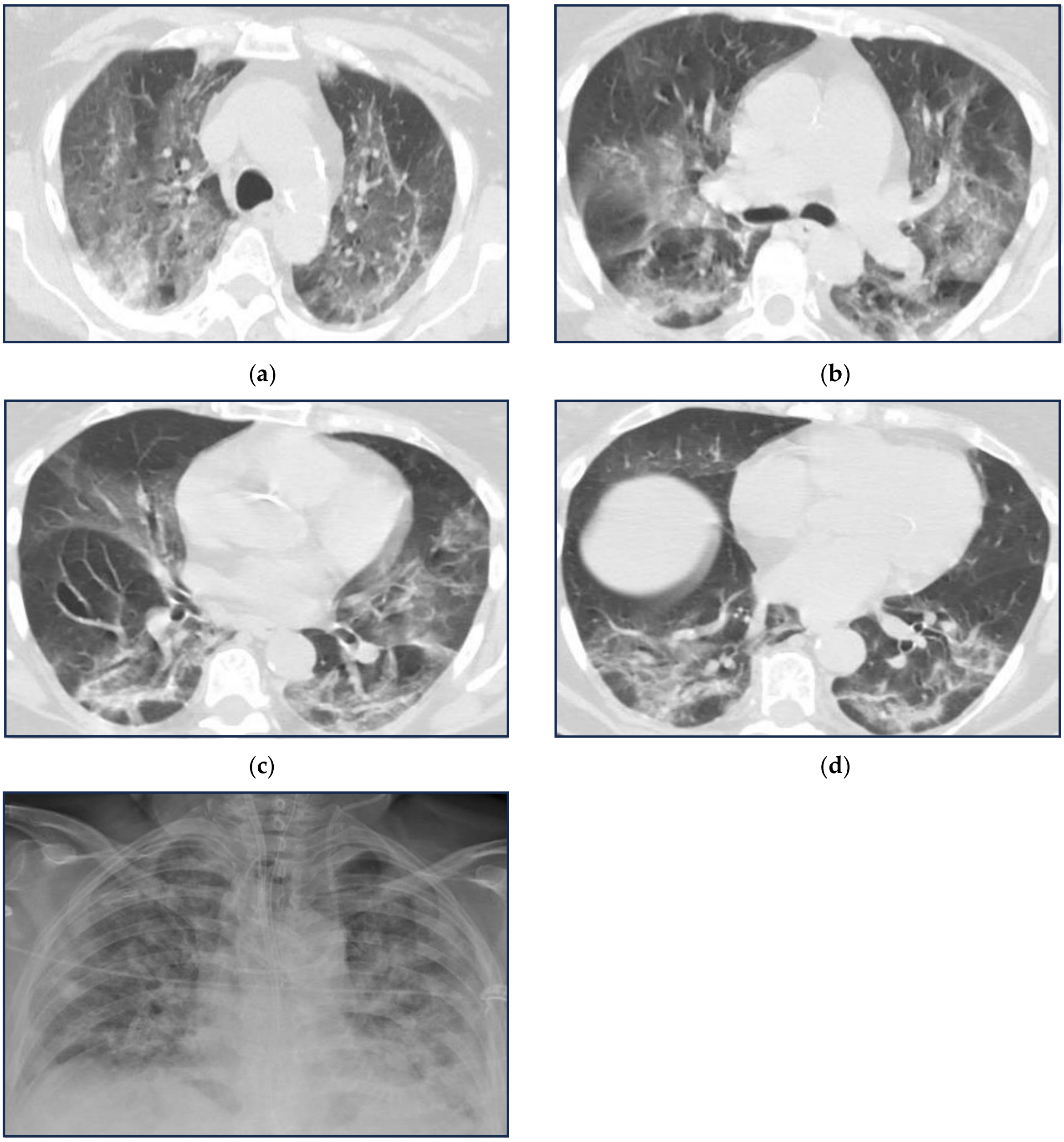

(**a**) (**b**) (**c**) (**d**) (**e**)

Supplementary Figure S1. Chest radiological imaging of the single SARS-CoV-2-positive 71 year old female patient with all 5 HHV reactivations detected at the same time. (**a**–**d**): computed tomography scan of the lungs on admission in ICU, showing multiple, bilateral, patchy and confluent ground glass opacities (GGO), with concomitant bilateral consolidative areas predominantly located in the subpleural region of the posterior segments of the lower lobes; the four images describe the lung pattern progressing from the apex to the base; (**e**) chest x-ray at the 21st day of hospital stay (still in ICU), showing multiple confluent parenchymal consolidative areas most evident at the lung bases, with pleural effusion at the left lung base; endotracheal tube, nasogastric tube and central venous catheter can also be observed. The patient eventually died 19 days later.
