# Supplementary material for: Clinical Features and Outcomes of Patients with Full Spectrum of COVID-19 Severity and Concomitant Herpesvirus Reactivation
Source: Microorganisms. 2025 May 27;13(6):1221. doi: 10.3390/microorganisms13061221 (PMC12195333; doi:10.3390/microorganisms13061221)
Supplement: Supplementary file 1 [file microorganisms-13-01221-s001.zip › Supplementary Table S1.pdf]

Supplementary Table S1. Comparison of the prevalence of reactivation of herpesviruses in different subgroups of SARS-CoV-2-negative and positive subjects in the study population (*n* = 153), with focus on more severe disease stages. Data are presented as frequencies (%). Bold values denote statistical significance at the $p < 0.05$ level. Analyzed subgroups were created by merging one or more of the following patient categories: (**1**) SARS-CoV-2-negative subjects (*n* = 51); (**2**) asymptomatic-to-mild SARS-CoV-2-positive subjects (*n* = 34) who underwent hospital discharge; (**3**): SARS-CoV-2-positive subjects with moderate disease (*n* = 32) and requiring low-to-medium intensity care; (**4**) SARS-CoV-2-positive subjects with severe-to-critical disease (*n* = 36) and who were admitted to ICU.

	HSV-1	p	HCMV	p	EBV	p	HHV-6	p	HHV-7	p
**1+2 (*n* = 85) vs. 3+4 (*n* = 68)**	4 (5) vs. 24 (35)	**< 0.001**	2 (2) vs. 8 (12)	**0.019**	18 (10) vs. 31 (46)	**0.003**	18 (10) vs. 40 (59)	**< 0.001**	34 (40) vs. 44 (65)	**0.003**
**1+2+3 (*n* = 117) vs. 4 (*n* = 36)**	12 (10) vs. 16 (44)	**< 0.001**	2 (2) vs. 8 (22)	**< 0.001**	32 (27) vs. 17 (47)	**0.002**	38 (32) vs. 20 (56)	**0.018**	52 (44) vs. 26 (72)	**0.008**
**1 (*n* = 51) vs. 4 (*n* = 36)**	1 (2) vs. 16 (44)	**< 0.001**	2 (4) vs. 8 (22)	**0.014**	9 (18) vs. 17 (47)	**0.006**	11 (22) vs. 20 (56)	**0.002**	20 (39) vs. 26 (72)	**0.004**
**2 (n = 34) vs. 4 (*n* = 36)**	3 (9) vs. 16 (44)	**< 0.001**	0 (0) vs. 8 (22)	**0.005**	9 (26) vs. 17 (47)	0.088	7 (21) vs. 20 (56)	**0.003**	14 (41) vs. 26 (72)	**< 0.001**
**3 (*n* = 32) vs. 4 (*n* = 36)**	8 (25) vs. 16 (44)	0.094	0 (0) vs. 8 (22)	**0.006**	14 (44) vs. 17 (47)	0.811	20 (62) vs. 20 (56)	0.627	18 (56) vs. 26 (72)	**0.001**
**2+3 (*n* = 66) vs. 4 (*n* = 36)**	11 (17) vs. 16 (44)	**0.002**	0 (0) vs. 8 (22)	**< 0.001**	23 (35) vs. 17 (47)	0.289	27 (41) vs. 20 (56)	0.212	32 (48) vs. 26 (72)	**0.023**

Abbreviations: Epstein-Barr virus (EBV); human cytomegalovirus (HCMV); human herpesvirus (HHV); herpes simplex virus (HSV); intensive care unit (ICU).
