# Supplementary material for: Clinical Features and Outcomes of Patients with Full Spectrum of COVID-19 Severity and Concomitant Herpesvirus Reactivation
Source: Microorganisms. 2025 May 27;13(6):1221. doi: 10.3390/microorganisms13061221 (PMC12195333; doi:10.3390/microorganisms13061221)
Supplement: Supplementary file 1 [file microorganisms-13-01221-s001.zip › Supplementary Table S2.pdf]

Supplementary Table S2. Comparison of the cycle threshold (Ct) values of SARS-CoV-2 RT-PCR in the subjects with and without HHV reactivation(s). Data are presented as median (range).

**Viral reactivation(s)**	**Ct of SARS-CoV-2 RT-PCR (patient *n* = 102)**		
	**HHV reactivation**	**No HHV reactivation**	**p**
HSV-1	28 (24-37) [1]	28 (20-33)	0.477
HCMV	32 (27-40) [1]	28 (21-33)	0.103
EBV	25 (20-32) [1]	28 (24-34)	0.384
HHV-6	27 (25-33) [1]	28 (20-33)	0.555
HHV-7	28 (22-33) [1]	27 (21-33)	0.453
≥1 any reactivation	28 (24-33) [1]	23 (16-35)	0.186
≥2 any reactivations	27 (22-33) [1]	28 (20-33)	0.944
≥3 any reactivations	27 (20-34) [1]	28 (22-33)	0.728

Abbreviations: cycle threshold (Ct); Epstein-Barr virus (EBV); human cytomegalovirus (HCMV); human herpesvirus (HHV); herpes simplex virus (HSV); real-time reverse transcriptase polymerase chain reaction (RT-PCR); [1] for the relative sample size of each category please refer to Table 3.
